# Microfragmented adipose tissue is associated with improved ex vivo performance linked to HOXB7 and b-FGF expression

**DOI:** 10.1186/s13287-021-02540-1

**Published:** 2021-08-28

**Authors:** Giulia Casari, Elisa Resca, Andrea Giorgini, Olivia Candini, Tiziana Petrachi, Maria Serena Piccinno, Elisabetta Manuela Foppiani, Lucrezia Pacchioni, Marta Starnoni, Massimo Pinelli, Giorgio De Santis, Filippo Selleri, Fabio Catani, Massimo Dominici, Elena Veronesi

**Affiliations:** 1 Department of Medical and Surgical Sciences for Children and Adults, University-Hospital of Modena and Reggio Emilia, Modena, Italy; 2 Rigenerand srl, Medolla, Modena, Italy; 3 Technopole Mario Veronesi, Mirandola, Modena, Italy; 4 Department of Orthopaedic and Traumatology, University-Hospital of Modena and Reggio Emilia, Modena, Italy; 5 Department of Pediatrics, Emory University, Atlanta, USA; 6 Division of Plastic Surgery, Department of General Surgery and Surgical Specialties, University-Hospital of Modena and Reggio Emilia, Modena, Italy

**Keywords:** Lipogems, MSC, Adipose tissue, Osteoarthritis, Microfragmentation

## Abstract

**Introduction:**

Adipose tissue (AT) has become a source of mesenchymal stromal/stem cells (MSC) for regenerative medicine applications, in particular skeletal disorders. Several enzymatic or mechanical procedures have been proposed to process AT with the aim to isolate cells that can be locally implanted. How AT is processed may impact its properties. Thus, we compared AT processed by centrifugation (C-AT) to microfragmentation (MF-AT). Focusing on MF-AT, we subsequently assessed the impact of synovial fluid (SF) alone on both MF-AT and isolated AT-MSC to better understand their cartilage repair mechanisms.

**Materials and methods:**

MF-AT and C-AT from the same donors were compared by histology and qRT-PCR immediately after isolation or as ex vivo cultures using a micro-tissue pellet system. The in vitro impact of SF on MF-AT and AT-MSC was assessed by histological staining and molecular analysis.

**Results:**

The main AT histological features (i.e., increased extracellular matrix and cellularity) of the freshly isolated or ex vivo-cultured MF-AT persisted compared to C-AT, which rapidly deteriorated during culture. Based on our previous studies of HOX genes in MSC, we investigated the involvement of Homeobox Protein HOX-B7 (HOXB7) and its target basic Fibroblast Growth Factor (bFGF) in the molecular mechanism underlying the improved performance of MF-AT. Indeed, both these biomarkers were more prominent in freshly isolated MF-AT compared to C-AT. SF alone preserved the AT histological features of MF-AT, together with HOXB7 and bFGF expression. Increased cell performance was also observed in isolated AT-MSC after SF treatment concomitant with enhanced HOXB7 expression, although there was no apparent association with bFGF.

**Conclusions:**

Our findings show that MF has a positive effect on the maintenance of AT histology and may trigger the expression of trophic factors that improve tissue repair by processed AT.

**Supplementary Information:**

The online version contains supplementary material available at 10.1186/s13287-021-02540-1.

## Introduction

Adipose tissue (AT) is a connective tissue composed of different cell types (e.g., adipocytes, pre-adipocytes and endothelial, hematopoietic, and stromal cells). Its main functions are mechanical protection, storage and release of energy, regulation of homeostasis and the secretion of adipokines [1–3]. Because of its availability as a biocompatible filler and its integration capacity into implantation sites, AT has been increasingly used in clinical procedures in autologous settings [4, 5]. Autologous grafted AT promotes tissue regeneration due to the presence of adipose tissue-derived mesenchymal stromal or stem cells (AT-MSC) within the so-called stromal vascular fraction (SVF) [6]. Several evidences report a significant positive clinical outcome such us in plastic surgery in the treatment of ulcers, burns, scars, soft tissue augmentation [5, 7–9]. The application of AT-MSC was not confined to plastic surgery but was also extended to cardiac, rheumatoid, gastro-intestinal and musco-skeletal fields, etc [10–12]. For this reason, different enzymatic and mechanical procedures have been established for processing AT to isolate cells that can be locally implanted [13, 14].

The standard harvesting procedure has been the Coleman technique, which uses liposuction followed by a centrifugation separation step to recover adipocytes and SVF for re-engraftment into the patient [4]. Although centrifuged AT (C-AT) has applications in the field of plastic and reconstructive surgery, pre-clinical and clinical studies have identified some limitations to this method, such as the appearance of fibrotic tissue after transplantation and partial graft reabsorption [15, 16]. Because how AT is processed may impact its properties, innovative systems of AT harvesting and processing have been developed [17]. New non-enzymatic methods can obtain a fat tissue derivative highly enriched in pericyte-like elements by mild mechanical forces from human lipoaspirates [14]. Among these methods, the microfragmentation AT (MF-AT) procedure can provide minimally manipulated ready-to-use AT-derived product by mechanical forces in the absence of enzymatic digestion. This technology processes lipoaspirates from patients by progressive size reduction of AT clusters through two filters and five stainless steel marbles [14]. The processed AT can be used in several tissue regeneration approaches, such as general surgery [8], plastic reconstructive and aesthetic surgery [18], oral-maxillofacial surgery and orthopaedic surgery for patients affected by osteoarthritis [19–21].

The rationale for using MF-AT in regenerative medicine is that this process maintains viable pericytes and AT-MSC within a preserved stromal vascular niche [14]. In osteoarthritis, MF-AT technology generates autologous tissue that can then be engrafted into the joint of the patient during a one-step surgery. Preliminary data (Level IV studies, case series) showed a clinically relevant reduction in pain, improved knee function and increased articular cartilage thickness even after two years of follow-up [22–25]. However, the mechanisms underlying the beneficial effects of MF-AT are not clearly understood [22, 26]. It is unclear what occurs in knees of osteoarthritic patients after MF-AT intra-articular injection. The cells themselves may be incorporated into the damaged tissue and differentiate into chondrocytes, or MF-AT may activate reparative mechanisms through specific pathways that influence resident chondrocyte repair, inflammation or both processes. In our previous studies, we showed how genes belonging to the HOXB family are associated with adipose and bone marrow MSC performance (i.e., osteogenesis and chondrogenesis) in association with increased basic Fibroblast Growth Factor (bFGF) [27, 28]. In those studies, the role of Homeobox Protein HOX-B7 (HOXB7) as a regulator of skeleton homeostasis was established, suggesting that HOXB7 can affect MSC through a bFGF-mediated autocrine loop [27]. It is known that bFGF promotes self-renewal and proliferation while inhibiting cellular senescence and ageing [29]. Tasso et al. [30] have demonstrated that AT-MSC stimulated in vitro by bFGF may secrete proteins involved in immune and inflammatory responses, wound healing and chemotaxis. When implanted in vivo, these cells can indirectly trigger wound healing and endogenous regenerative mechanisms following tissue damage [30].

Osteoarthritis (OA) is a pathological condition resulting in cartilage degradation and bone damage [31]. A close relationship between cytokine expression and OA has been found [32, 33].

In fact, it was shown that interleukin-1 (IL-1) and tumour necrosis factor-alpha (TNF-alpha) can induce the production of interleukin-6 (IL-6) and interleukin-8 (IL-8) by synovial cells.

Synovial Fluid (SF) has biomechanical, metabolic, and regulatory functions. SF is normally a clear, straw-colored, viscous liquid [34]. A major component of SF composition is proteins such as cytokines and growth factors that are at relatively low concentrations in normal SF, and are markedly elevated in joint injury and disease. Cytokines may be categorized as pro- or anti-inflammatory according to their predominant tissue-specific effects. For example, in SF cytokines such as IL-1α, IL-1β, TNF-α, leukemia inhibitory factor (LIF), IL-6, IL-8, IL-17, and IL-18 play a pro-inflammatory effect, whereas IL-4, IL-10, and IL-13 exert and anti-inflammatory potential. Growth factors found in SF include TGF-β1 and insulin growth factor 1 (IGF-I) and have anabolic effects. Most important are also several binding proteins in SF and playing an important role in cell regulation [35].

**Fig. 1:**
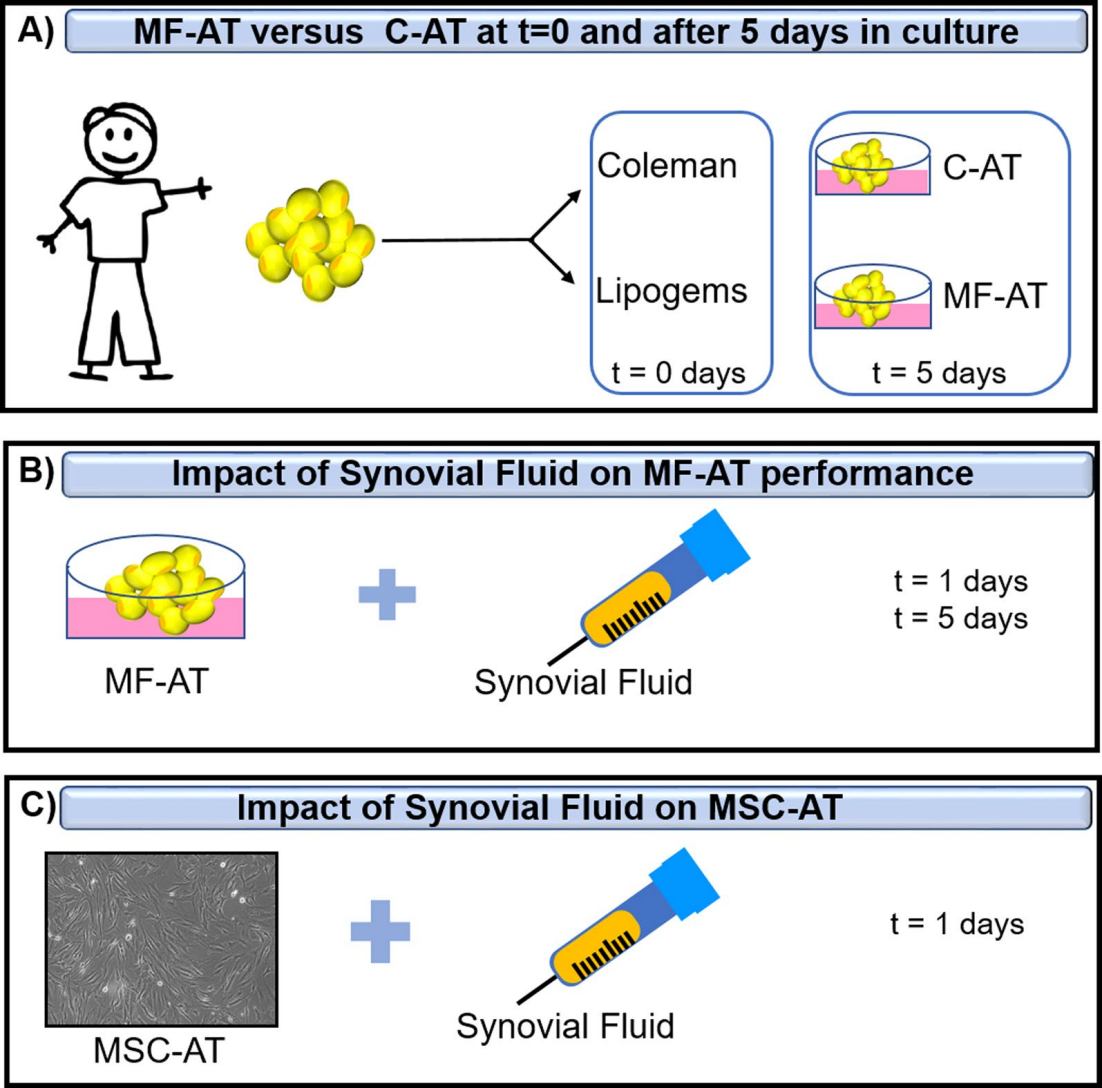
Overview of experiment. **a** AT has been harvested and processed according to Lipogems or Coleman techniques to obtain, respectively, MF-AT and C-AT. Specimens were processed for histological assays and qRT-PCR (*t* = 0, *t* = 5 days). **b** MF-AT were incubated with osteoarthritis SF and processed at 1 days and 5 days for histological assays and qRT-PCR. **c** Similarly, MSC-AT were stimulated with SF and analysed by viability assay (MTT), histology and qRT-PCR

In the current study, we compared the in vitro fates of MF-AT and C-AT using a micro-tissue system with or without pathologic (i.e., osteoarthritic) SF to artificially mimic the articular space and the processes happening in vivo after AT transplant. Histological and molecular analyses, including examination of changes in HOXB7 and bFGF expression, were used to investigate how the knee microenvironment can be positively influenced by processed AT (Fig. 1).

## Results

### Adipose tissue microfragmentation preserves the stromal fraction better than the Coleman technique

**Fig. 2:**
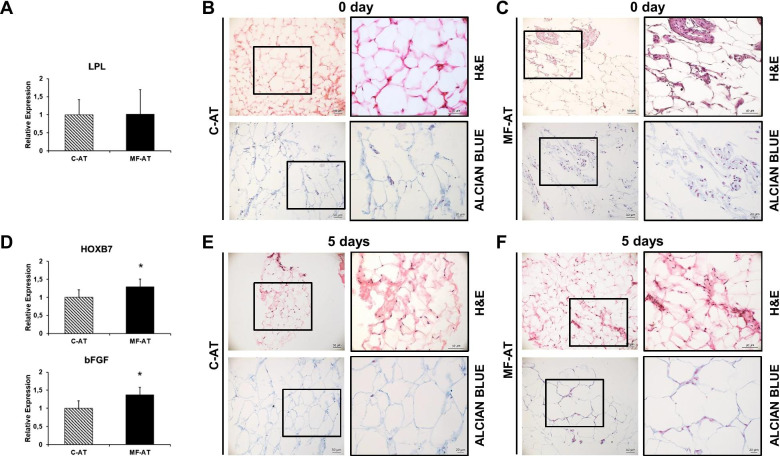
Comparison of histological staining and gene expression of MF-AT and C-AT. **a**, **d** qRT-PCR analysis of gene expression. LPL (**a**), HOXB7 and bFGF (**d**) mRNA expression levels were measured in freshly isolated MF-AT and C-AT (*p = 0.01 for HOXB7 and *p = 0.02 for bFGF, unpaired two-tailed Student’s t-test). Error bars, SEM. (**b**, **c**, **e**, **f**) H&E and Alcian Blue-Nuclear Fast Red staining of freshly isolated C-AT (**b**) and MF-AT (**c**) and their respective five-day cultures (**e**, **f**). Photomicrographs were acquired using an Axio Imager M2 (Zeiss) with EC Plan APOCHROMAT ×20/0.8 and EC Plan Neofluar ×40/0.75 objectives

Freshly isolated C-AT and MF-AT obtained from the same donors (*n* = 10) were analysed for basic AT features. The two groups had similar LPL mRNA levels, a typical mature AT marker [36], indicating that both procedures preserved the lipogenic nature of the specimens (Fig. 2a). Haematoxylin and eosin (H&E) staining confirmed that C-AT retained the typical histological properties of human fat, including mature adipocytes, a stromal fraction and blood cells (Fig. 2b and inset). MF-AT not only preserved these features but revealed enriched cellularity with a greater extracellular matrix (Fig. 2c and inset). The glycosaminoglycan nature of the observed tissues was confirmed by the intense Alcian Blue staining of the MF-AT samples only, demonstrating the presence of a more abundant stromal extracellular matrix after MF processing (Fig. 2b, c and insets).

### MF-AT preservation is associated with higher HOXB7 and bFGF levels

HOXB7 is downregulated in MSC during ageing and positively connected with the increased performance of these cells [27, 28]. To investigate the distinct behaviour of the differentially processed AT, HOXB7 expression was evaluated. Significantly higher levels of HOXB7 were observed in MF-AT compared to C-AT (Fig. 2d, upper graph; *p* = 0.01). Because bFGF is a known transcriptional target of HOXB7 involved in tissue repair and regeneration [27, 37], we determined the expression of this factor in the MF-AT and C-AT samples. Interestingly, freshly processed MF-AT had greater bFGF expression compared to C-AT (Fig. 2d, lower graph; *p* = 0.02), indicating that the higher HOXB7 and bFGF levels may account for the better MF-AT preservation.

### MF-AT, not C-AT, retains higher cellularity and abundant stroma

Isolated samples were cultured in a maintenance medium to assess the long-term persistence of AT structure and functionality. After 5 days, progressive deterioration of the C-AT structures was observed with a massive loss of cellularity in the mature adipocytes and stromal compartment (Fig. 2e and inset). Moreover, Alcian Blue staining was not detected (Fig. 2e and inset). In contrast, 5-day-old MF-AT cultures displayed a better-preserved adipose cell compartment with enriched cellularity (Fig. 2f and inset) and positive Alcian Blue staining of the stromal fraction (Fig. 2f and inset). The HOXB7 and bFGF expression levels after the 5-day-old cultures did not significantly differ between C-AT and MF-AT (data not shown). Collectively, these data suggest that MF-AT performed better than C-AT overall; however, this finding was not associated with a persistent molecular pattern.

### Osteoarthritic SF-stimulated MF-AT preserved ex vivo and is associated with increased proliferation

**Fig. 3:**
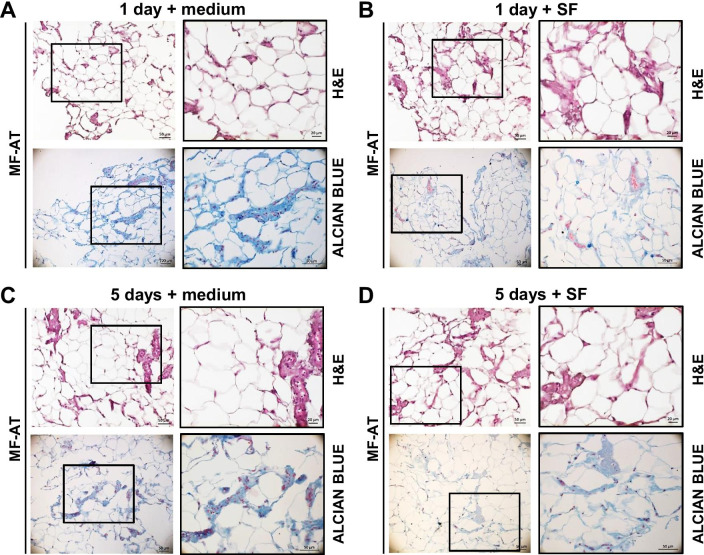
H&E and Alcian Blue staining of MF-AT treated with SF. H&E and Alcian Blue-Nuclear Fast Red staining of MF-AT following treatment with SF or maintenance medium for 1 (**a**, **b**) or 5 (**c**, **d**) days. Photomicrographs were acquired using an Axio Imager M2 (Zeiss) with EC Plan APOCHROMAT ×20/0.8 and EC Plan Neofluar ×40/0.75 objectives

**Fig. 4:**
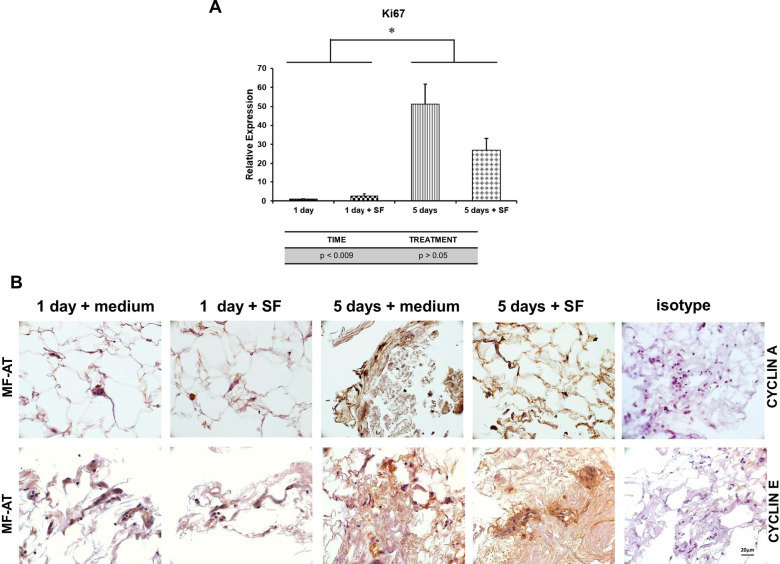
Ki67 expression in MF-AT treated with SF. qRT-PCR analysis of Ki67 mRNA expression levels in MF-AT evaluated at 1 or 5 days after treatment with SF or maintenance medium (*p = 0.000001, two-way ANOVA). Error bars, SEM

Based on the known regenerative properties of MF-AT, we developed an ex vivo preclinical model to mimic what may take place in the knees of patients affected by osteoarthritis after MF-AT transplantation. This model was used to verify whether pathologic SF has a detrimental impact on MF-AT performance. 1-day and 5-day micro-tissue pellet cultures were compared after seeding MF-AT (*n* = 3) in SF from osteoarthritic patients or control maintenance medium. H&E staining of the 1-day and 5-day cultures was used to evaluate the biological impact of SF on MF-AT. This staining revealed viable tissue and the preservation of typical AT architecture containing mature adipocytes and an SVF (Fig. 3a–d). Alcian Blue staining confirmed the conserved AT glycosaminoglycan nature of the cultures at both time points and culture conditions (Fig. 3a–d). There were no substantial differences between the SF and control medium cultures. The levels of Ki67 (proliferation marker) were analysed to evaluate the possible effect of SF on the proliferation of the MF-AT ex vivo cultures. A statistically significant increase in Ki67 levels was noticed between days 1 and 5 in culture (*p* = 0.000001), indicating that the osteoarthritic SF was able to maintain MF-AT proliferation without the addition of growth supplements (*p* = 0.7 vs control medium, not statistically significant) (Fig. 4a).

In order to assess the impact of SF, we evaluated the expression of Cyclin A and Cyclin E as key regulator of cell cycle. As shown in Fig. 4b, SF didn’t alter Cyclin A and E expression both after 1 day and 5 days of cell culture, confirming that SF didn’t impact the cell proliferation. After 5 days of stimulation, we detected an important increase in both cyclins’ expression, strongly confirming the maintained viability of organ culture. These data demonstrated that pathologic SF does not affect MF-AT performance ex vivo.

The importance of the impact of SF on cell proliferation was evaluated also after one day of stimulation in a Lipogems-derived MSC. We performed BrdU staining in order to detect and to quantify the quote of the cells in S phase of cell cycle. SF stimulation showed approximately twofold increased fluorescence compared to untreated cells, as reported in figure S1.

### Preservation of the osteoarthritic SF-stimulated micro-tissue system is associated with high HOXB7 and bFGF expression levels

**Fig. 5:**
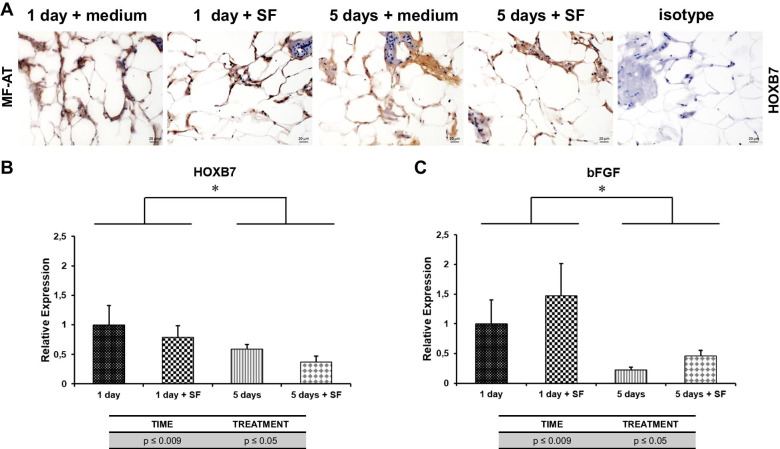
HOXB7 and bFGF expression in MF-AT treated with SF. **a** HOXB7 protein expression in MF-AT by IHC. Analysis was performed 1 or 5 days after treatment with SF or maintenance medium. Photomicrographs were acquired using an Axio Imager M2 (Zeiss) with an EC Plan Neofluar ×40/0.75 objective. **b**, **c** qRT-PCR analysis of HOXB7 (**b**) and bFGF (**c**) mRNA expression levels in MF-AT 1 or 5 days after treatment with SF or maintenance medium (*time *p* = 0.0003 and treatment *p* = 0.04 for HOXB7; *time *p* = 0.000005 and treatment *p* = 0.03 for bFGF; two-way ANOVA). Error bars, SEM

HOXB7 expression was analysed by immunohistochemistry (IHC) and quantitative real time-PCR (qRT-PCR) to assess the role of SF in preserving the performance of MF-AT. As shown in Fig. 5a, HOXB7 staining was observed after 1 day of culture and increased at day 5 after SF stimulation (Fig. 5a). In contrast, HOXB7 mRNA expression decreased from days 1 to 5 (*p* = 0.0003). SF stimulation further decreased HOXB7 mRNA levels compared to the control medium (*p* = 0.04) (Fig. 5b). The mRNA levels of the HOXB7 target bFGF were also evaluated to analyse the downstream effects of HOXB7 on MF-AT. The bFGF mRNA levels significantly decreased (time *p* = 0.000005) from days 1 to 5 in culture. Stimulation with SF increased bFGF mRNA levels at both time points (*p* = 0.03), confirming a role for SF in promoting tissue repair and regeneration (Fig. 5c).

These data suggested that HOXB7 and bFGF induced by osteoarthritic SF might account for MF-AT preservation.

We performed In-Cell western in order to quantify the intracellular relative protein levels of HOXB7 and bFGF.

In this system, SF stimulation decreases the levels of HOXB7 protein at 1 and 5 days of treatment (Fig. S2A). bFGF protein decreased after 5 days of culture. Indeed, at 1 day, SF stimulation, not significantly impact on bFGF expression (Fig. S2B).

### Synovial fluid alters AT-MSC morphology, HOXB7 expression and metabolic activity

**Fig. 6:**
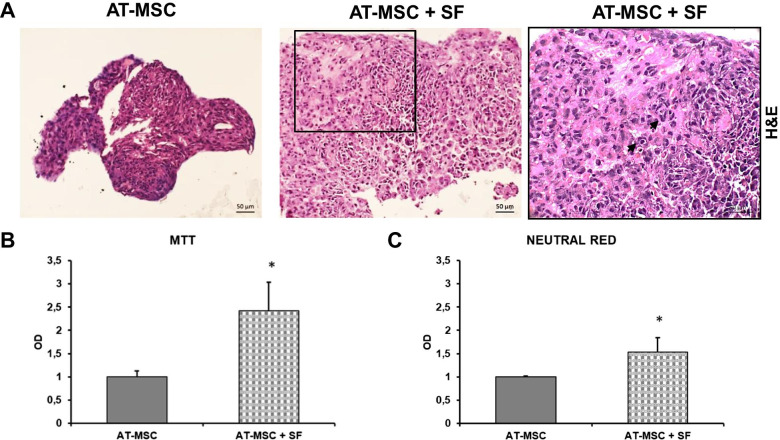
H&E staining and proliferative rate of 3D AT-MSC cultures after SF treatment. **a** H&E staining of 3D AT-MSC cultures 1 day after treatment with SF or maintenance medium. Photomicrographs were acquired using an Axio Imager M2 (Zeiss) with an EC Plan APOCHROMAT ×20/0.8 objective. Arrows (inset): large cytoplasmic cells resembling chondrocytes. **b**, **c** Proliferative rates of 2D AT-MSC cultures determined by MTT (**b**) and Neutral Red (**c**) assays 1 day after treatment with SF or maintenance medium (*p = 0.01 for both assays, unpaired two-tailed Student’s t-test). Error bars, SD

To better define the effect of SF on AT-derived cells, we developed 2D and 3D pellet culture in vitro models of isolated AT-MSC treated for 24 h with SF from patients with osteoarthritis or control maintenance medium. H&E staining of the 3D AT-MSC pellet treated with SF revealed increased cellularity and a higher amount of extracellular matrix compared to the control (Fig. 6a). Interestingly, AT-MSC treated with SF contained a mix of spindle and round-shaped cells with a large cytoplasm resembling chondrocytes (arrows in Fig. 6a inset). MTT and Neutral Red metabolic assays were performed on 2D cultures to further compare SF and the maintenance medium. Both assays showed a statistically significant increase in AT-MSC viability after SF stimulation compared to the control medium (MTT *p* = 0.01; Neutral Red *p* = 0.01) (Fig. 6b, c). Collectively, these data indicated how SF could modify AT-MSC morphology and viability.

**Fig. 7:**
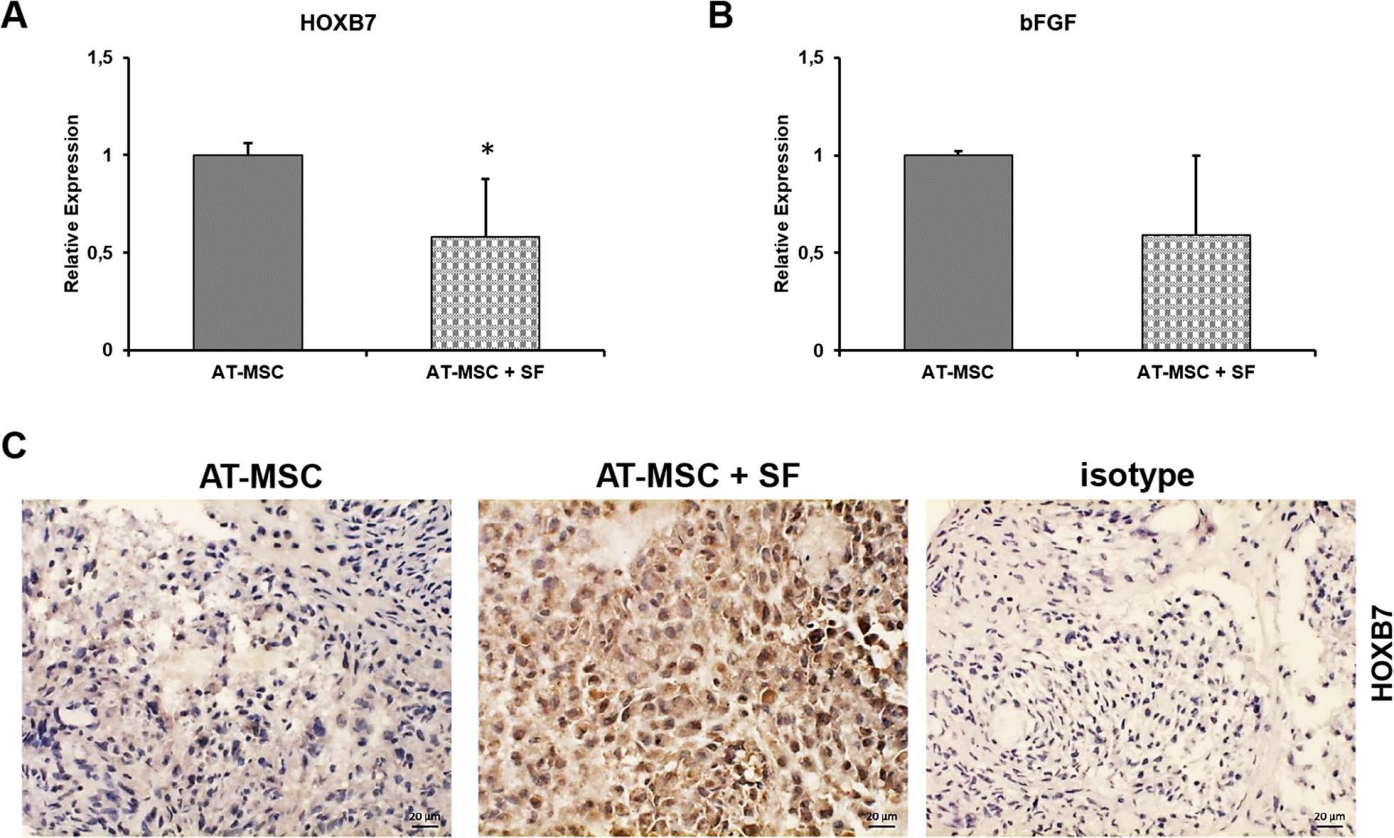
HOXB7 and bFGF expression in 3D AT-MSC cultures treated with SF. **a**, **b** qRT-PCR analysis of HOXB7 (**a**) and bFGF (**b**) mRNA expression levels in 3D AT-MSC cultures 1 day after treatment with SF or maintenance medium (*p = 0.002 for HOXB7, unpaired two-tailed Student’s t test). Error bars, SD. (**c**) HOXB7 protein expression in 3D AT-MSC cultures by IHC. Analysis was performed 1 day after treatment with SF or maintenance medium. Photomicrographs were acquired using an Axio Imager M2 (Zeiss) with an EC Plan Neofluar ×40/0.75 objective

HOXB7 and bFGF expression were also analysed to determine the effect of SF on the performance of 3D AT-MSC cultures. HOXB7 and bFGF mRNA levels decreased after treatment with SF for 24 h (*p* = 0.002 vs control; Fig. 7a, b). However, HOXB7 protein levels increased after the 24 h SF treatment (Fig. 7c). Collectively, these data indicated that SF influenced isolated AT-MSC performance by modulating HOXB7/bFGF pathways.

## Discussion

The use of AT as a source of MSC for regenerative medicine applications (e.g., skeletal disorders) is a rapidly growing area of research. The current study clarified some aspects of AT behaviour ex vivo and demonstrated that better preserved AT features are obtained using microfragmentation rather than centrifugation during ex vivo manipulation. A positive outcome in osteoarthritis treatment has been reported [10, 11, 38] MF-AT had surprisingly higher HOXB7 and bFGF expression in a pathologic SF environment, suggesting that these features are possible players in the beneficial impact on joint homeostasis. To investigate which AT processing method better preserves AT histology and performance and promotes regenerative mechanisms, two different methods (MF vs. C) were compared. The MF-AT cultures in a maintenance medium were well preserved with higher cellularity and stromal extracellular matrix compared to C-AT. The increase in the cellular and stromal compartments, rich in Glycosaminoglycan, in MF-AT compared to C-AT was consistent with the findings of Bosetti et al. [26] on chondrogenic organ cultures from lipoaspirates.

In our study, the better performance of MF-AT was originally investigated by analysing HOXB7 and bFGF expression. Indeed, it is known that HOXB7 is downregulated by high levels of miR196 in aged MSC, thus regulating ageing rather than proliferation, skeleton formation and homeostasis in the post-natal skeleton [27]. The enhanced HOXB7 mRNA levels of freshly isolated MF-AT could play an important role in the mechanisms underlying the clinical success of MF-AT through specific molecular pathways. Increased HOXB7 expression in human AT was previously observed in human abdominal subcutaneous AT depots compared to gluteal depots [39]. However, its expression in differentially processed AT was not previously reported. bFGF mRNA levels also increased in the freshly prepared MF-AT. It is known that bFGF indirectly promotes wound healing and immune and inflammatory responses, thus triggering endogenous regenerative mechanisms against tissue damage [30]. Therefore, the MF-AT regenerative potential may be linked to the activation of HOX pathways by mechanical stimuli that can upregulate anti-inflammatory genes and, in parallel, downregulate TNF-α in lipoaspirates [40]. In addition, it has been reported that mechanically stimulated MSC express chondrogenic genes [41]. Thus, it may be that mechanical stimulation is crucial for the enhanced expression of HOX genes and should be further investigated.

We developed an ex vivo model that mimics the osteoarthritic setting after MF-AT transplant. SF is a key component of the knee microenvironment that promotes cartilage lubrification and reduces attrition. Significant changes occur during osteoarthritis because of direct alterations in SF and indirect changes in the surrounding synovial tissues [35]. To understand how the pathologic changes in SF can influence AT survival, we used SF as a culture medium. The organotypic AT cultures developed by Bosetti et al. [26] and rat AT fragments incorporated in collagen type I described by Toda et al. [42] have been used to study AT regenerative mechanisms [26, 42]. However, to our knowledge, an organotypic MF-AT culture using SF alone has not been previously generated. This relatively simple approach can preserve MF-AT architecture for up to 5 days in culture, even without additional growth factors. Thus, this approach overcomes the current limitations of organotypic cultures that have never survived more than 48 h [43], allowing a longer observation time for deeper investigation.

In this model, basic cell features were evaluated, such as the proliferation marker Ki67, and cell cycle regulators Cyclin A and Cyclin E [44–46]. Ki67 was expressed at low levels at day 1, possibly due to the quiescent state of cells during the early phases of culture. Increased Ki-67 was observed after 5 days in culture, confirming that the short half-life of cultured tissue can be overcome. Similarly, expression of Cyclin A and Cyclin E confirm this trend. Furthermore, HOXB7 was expressed in the MF-AT at all time points with or without SF stimulation. The high proliferation rate of MF-AT at 5 days might be related to the high HOXB7 expression, as shown by our group for tissue-isolated MSC [27, 28].

In this study, we observed an inverse relationship between HOXB7 mRNA and protein expression that requires further investigation. These results may be explained by negative feedback mechanisms on HOXB7 transcription that are triggered by high HOXB7 protein levels. Such mechanisms have been described for other transcription factors controlling stem cell pluripotency and self-renewal [47]. Therefore, we hypothesize that a mechanism similar to that previously described for Oct4, Nanog and FoxD3 [47] may be necessary to maintain a proper balance of HOXB7 expression levels.

The increased HOXB7 described in this study may, in part, explain the enhanced cellularity and stromal compartment in MF-AT compared to C-AT. Because HOXB7 effects can be mediated by bFGF, an important HOXB7 transcriptional target [27, 37] expressed by human pericytes [48] and adipocytes [49], its mRNA levels were investigated in MF-AT following SF treatment. SF increased bFGF mRNA levels in 1-day and 5-day cultures, suggesting that SF promotes the activation of regenerative mechanisms. The trends of HOXB7 and bFGF mRNA expression in MF-AT cultured with SF were opposite each other, suggesting that bFGF levels might be upregulated not only by HOXB7 but also by cytokines contained in the SF. Our ex vivo model has highlighted the different biological aspects of MF-AT for regenerative approaches; however, the precise role of SF in the preservation of AT in knee osteoarthritis has not been completely defined due to the limitations of organotypic ex vivo cultures and the complexity of the pathology.

Because of these limitations, we also developed a simplified in vitro model to evaluate the impact of SF on isolated AT-MSC. This model showed that, after one day of treatment with osteoarthritic SF, the AT-MSC proliferation rate significantly increased compared to control conditions, and their morphology became similar to chondroblasts. Indeed, treatment of 3D cultures of horse BM-MSC with autologous SF in culture medium for 26 days induces high expression of both proteoglycans and type II collagen, indicating that SF promotes chondrogenic differentiation of MSC and supporting their use to treat cartilage defects [50]. In our model, there was no effective chondrogenic differentiation of AT-MSC after treatment with SF, which might indicate that the time points of our analysis were too early; however, the modified morphology suggested an initial propensity for this commitment. Moreover, AT-MSC treated by SF in the absence of culture medium cannot be cultured for a long time, which is required for standard chondrogenic differentiation assays [28].

SF significantly enhanced HOXB7 expression by IHC, suggesting that SF may help to prevent MSC ageing and preserve their regenerative potential. A previous study of HOXB7 expression in MSC suggested that, in vitro, the upregulation of this gene in AT-MSC increased the cell proliferation rate, decreased senescence and improved osteogenesis, resulting in MSC cellular reprogramming [27]. This finding is consistent with our observations in the in vitro model, where high HOXB7 levels likely accounted for the increased AT-MSC proliferation rate after SF stimulation. To investigate the downstream effect of HOXB7 following SF treatment, we examined bFGF mRNA levels and found a decreasing trend that did not reach statistical significance. Thus, SF did not affect the transcription of this gene in AT-MSC, suggesting that the increase in bFGF levels observed after SF treatment in the ex vivo model may be related to a combination of tissue microfragmentation and SF treatment and not to the SF effects on AT-MSC alone.

While several aspects of the regenerative potential of MF-AT need to be clarified, our study suggests that the mechanical procedure, MF, used to obtain adipose specimens preserves MF-AT histology in association with HOXB7 gene expression and trophic factors capable of preserving AT itself and, potentially, contributing to tissue repair. A better understanding of MF-AT biology is a pre-requisite to a better understanding of the substantial benefit of MF-AT transplantation in patients [51]. Our study presents a novel procedure that could potentially enhance the therapeutic profile of AT to achieve a more favourable prognosis.

## Conclusion

In conclusion, our study better clarifies the biology of MF-AT showing the expression of trophic factors such as bFGF improving the tissue viability. Cell model with MF-AT and SF confirms that inflammatory microenvironment didn’t decrease expression of those trophic factors. Our finding may highlight the potential benefit of MF-AT treatment of inflammatory diseases achieving a more favourable prognosis.

## Materials and methods

The main idea of the manuscript is summarized in Fig. 1.

### Patients

Lipoaspirates used to compare C-AT and MF-AT processing were performed from the abdominal site of patients (*n* = 10) referred to Department of Medical and Surgical Sciences for Children & Adults, University of Modena and Reggio Emilia. Lipoaspirates used to evaluate SF impact on MF-AT and AT-MSC were obtained from the abdomens of patients (*n* = 3) affected by knee osteoarthritis referred to the Departments of Orthopaedic Surgery and Plastic, Reconstructive and Aesthetic Surgery, University of Modena and Reggio Emilia. SF were harvested from the knees of patients (*n* = 8) affected by osteoarthritis referred to the Department of Orthopaedic Surgery, University of Modena and Reggio Emilia. AT-MSC were isolated from lipoaspirates of patients (*n* = 2) who underwent plastic breast reconstructive surgery following mastectomy. All procedures were performed with informed consent and approval by the Local Ethics Committee n. 254. The specimens were tested for the presence of HIV (1 and 2), hepatitis C virus, hepatitis B virus and cytomegalovirus, and all specimens were negative for all these pathogens.

### AT harvesting and processing

The surgical procedure for AT harvesting was performed under local anaesthesia and sedation. After the skin incision, the donor abdominal area was infiltrated using a blunt cannula filled with anaesthetic solution (100 mL saline, 10 mL 7.5 mg/mL levobupivacaine, 20 mL 10 mg/mL mepivacaine and 0.5 mL 1 mg/mL epinephrine). AT was harvested through the same incision with a blunt cannula. The cannula used for sampling was connected to a syringe that was progressively filled. The mean volume of lipoaspirate collected from each patient was 60 mL. MF was performed according to the method of Randelli et al. [52] on 30 mL lipoaspirate using a Lipogems device and yielded 10 mL MF-AT. The remaining lipoaspirate (30 mL) was processed by centrifugation using the Coleman technique [4]. The lipoaspirate was centrifuged at 3000 rpm for 3 min, resulting in three distinct layers: top layer, oil derived from fat cells; intermediate layer, adipocytes and SVF; bottom layer, blood cells, water and anaesthetic mixture. The intermediate layer (C-AT) was collected for subsequent experiments [4, 53].

MF-AT and C-AT were cultured in maintenance medium, consisting of Minimum Essential Medium (α-MEM) without nucleosides (Gibco Life Technology), 2.5% Platelet lysate, 2 mM L-glutamine, 100 U/mL penicillin, 100 μg/mL streptomycin (all from Gibco Life Technology) and 0.2% heparin (Sigma-Aldrich), for 5 days at 37 °C and 5% CO_2_. The ex vivo model was generated by adding SF in the absence of culture medium to MF-AT for 1 or 5 days at 37 °C and 5% CO_2_. MF-AT cultured in maintenance medium were used as a negative control.

### AT-MSC culture and metabolic assays

AT-MSC were isolated from AT as previously described [54] and cultured at a density of 6000 cells/cm^2^ in maintenance medium at 37 °C and 5% CO_2_. Subsequently, AT-MSC were cultured in SF alone or maintenance medium for 1 day at 37 °C and 5% CO_2_. MTT and Neutral Red metabolic assays were performed as previously reported (UNI EN ISO 10993-5:2009). The in vitro model (pellet) was generated by culturing 3 × 10^5^ AT-MSC in maintenance medium in conical polypropylene centrifuge tubes (EuroClone) at 37 °C and 5% CO_2_. After 1 day of culture, the in vitro models were stimulated with SF, or maintenance medium as negative control, and cultured for an additional day.

### Histology and immunohistochemical analysis

Histology and immunohistochemical analysis were performed on MF-AT, C-AT and the in vitro models. Four-μm sections were obtained from formalin-fixed, paraffin-embedded samples. Histological analysis was performed using H&E (Sigma-Aldrich) and Alcian Blue-Nuclear Fast Red (Sigma-Aldrich) staining. IHC was performed as previously described [21]. Paraffin sections were stained with rabbit anti-human HOXB7 (1:40; Abnova), rabbit anti-human cyclin A (1:75; Santa Cruz) and rabbit anti-human Cyclin E (1:75; Santa Cruz). Staining was analysed using the Axio Imager M2 (Zeiss; EC Plan APOCHROMAT 20×/0.8 and EC Plan Neofluar 40×/0.75 objectives) and ZenPro software (Zeiss).

### qRT-PCR

Total RNA from MF-AT, C-AT and 3D AT-MSC cultures was harvested using TRIzol reagent, according to the manufacturer’s instructions (Invitrogen). cDNAs were generated with random hexamers using the RevertAid First Strand cDNA Synthesis Kit (Fermentas, Thermo Fisher Scientific). RNA and cDNA were quantified with a Beckman Coulter spectrophotometer. qRT-PCR was performed using Fast SYBR Green Master Mix (Applied Biosystems, Thermo Fisher Scientific) with the StepOne Real-Time PCR System (Applied Biosystems, Thermo Fisher Scientific). The PCR primers are listed in Table 1. β-actin was used for normalization. Measurements were performed in triplicate. Relative expression was determined using the ΔΔCt method [55].

**Table 1:** qRT-PCR primers

Gene	Primer sequence	Amplified length (bp)
*β Actin*	5′-ACCTTCTACAATGAGCTGCG-3′ (sense)	148
	5′-CCTGGATAGCAACGTACATGG-3′ (antisense)	
*LPL*	5′-GAAGACTCGTTCTCAGATGCC-3′ (sense)	145
	5′-GAATGGGATGTTCTCACTCTCG-3′ (antisense)	
*HOXB7*	5′-CCTGGATGCGAAGCTCAG-3′ (sense)	107
	5′-CGTCAGGTAGCGATTGTAGTG-3′ (antisense)	
*bFGF*	5′-ACCCTCACATCAAGCTACAAC-3′ (sense)	141
	5′-AAAAGAAACACTCATCCGTAA-3′ (antisense)	
*Ki67*	5′-GTCGTGTCTCAAGATCTAGCTTC-3′ (sense)	146
	5′-GTCATCTGCGGTACTGTCTTC-3′ (antisense)	

### Statistics

Data are presented as the mean ± SEM, except for the study of SF impact on AT-MSC in which data are presented as the mean ± SD. Comparisons of two groups were made with the two-tailed t-test using Microsoft Excel 2010. The study of SF impact on AT was analysed using two-way ANOVA (GraphPad Prism 7.04 software). Statistical significance was indicated by *p* < 0.05.

## Supplementary Information

**Additional file 1.** Supplementary Material and Method.

**Additional file 2. Fig. S1**: BrdU staining of Lipogems derived MSC after SF stimulation for 24 hours. SF stimulation showed approximately twofold increased fluorescence compared to untreated cells, resulting in 63,3% of positive cells when SF is added to cell culture versus 27.0% obtained in untreated cells. **Fig. S2**: In-Cell Western quantification of the relative intracellular level of HOXB7 (Fig S2A) and bFGF (Fig S2B) protein in Lipogems derived MSC after 1 and 5 days of stimulation with SF. At 1 day of SF stimulation, the level protein of HOXB7 decreases in a statistically significant manner. Indeed, at 5 days no significant effect on HOXB7 protein expression were observed when SF was added. Moreover, untreated cells decreased the levels of HOXB7 at 5 days of culture (p-value<0.05). SF, decreased the level of bFGF after 5 days of culture compared to 1 day of treatment (p-value<0.05). Moreover, bFGF protein decreases when cells are treated with SF for 5 days (p-value<0.05).

## Data Availability

Please contact author for data requests.

## Abbreviations

α-MEM: Minimum essential medium
AT: Adipose tissue
AT-MSC: Adipose tissue mesenchymal stem cell
bFGF: Basic fibroblast growth factor
BM-MSC: Bone marrow mesenchymal stem cell
C-AT: Coleman adipose tissue
cDNA: Complementary deoxyribonucleic acid
H&E: Haematoxylin & Eosin
IHC: Immunohistochemistry
Oct4: Octamer-binding transcription factor 4
HOX B7: Homeobox B7
MF-AT: Microfragmented adipose tissue
MSC: Mesenchymal stem cell
OA: Osteoarthritis
PCR: Polymerase chain reaction
RNA: Ribonucleic acid
SF: Synovial fluid
SVF: Stromal vascular fraction
