# Supplementary material for: Microfragmented adipose tissue is associated with improved ex vivo performance linked to HOXB7 and b-FGF expression
Source: Stem Cell Res Ther. 2021 Aug 28;12:481. doi: 10.1186/s13287-021-02540-1 (PMC8399787; doi:10.1186/s13287-021-02540-1)
Supplement: Supplementary file 1 — Additional file 1. Supplementary Material and Method. [file 13287_2021_2540_MOESM1_ESM.docx]

Supplementary Material and Method

M1) MF-AT Lipogems Derived MSC Isolation. MF-AT was suspended in culture media composed by aMEM supplemented with 2,5% of platelet lisate, 1% L-Glutamine, 0,2%Heparin and 1% P/S (X% v/v) and incubated at 37°C, 5%CO2, in MW6 (Greiner). Adherent fibroblastoid cells were detached using trypsin 0,01%/EDTA0,002%, seeded at 6000/cm2 and amplifying for 2 passages. Then, MF-AT Lipogems derived MSC were seeded at 6000/cm2 in T25 flask as well as in MW96 and promptly incubated with culture media or synovial fluid for 24 h and 5 days for BRDU and In-Cell Western assays.

M2) BrdU staining. BrdU Flow Kit (BD Pharmingen) was used for BrdU staining, in order to assess the role of SF on cell proliferation. Staining was performed according to manufacturer instruction. 10 µL of BrdU solution (1 mM BrdU in 1X DPBS) was added to each mL of tissue culture medium and cells were incubated overnight at 37°C/5%CO2. The day after, cells were detached, fixed and permeabilized with 100 µL of BD Cytofix/ Cytoperm Buffer per tube for 20 minutes on ice. 1 mL of 1X BD Perm/Wash Buffer was added and cells were centrifuged for 5 minutes at 300g. Supernatant was discared and cells were incubated with BD Cytoperm Permeabilization Buffer (100 µL per tube) for 10 minutes on ice. Cells were washed with1 mL of 1X BD Perm/Wash Buffer and centrifuged. After supernatant removal, cells were re-fixed with BD Cytofix/ Cytoperm Buffer (100 µL per tube) for 5 minutes on ice. After wash, cells were treated with DNase (diluted to 300 µg/mL in DPBS, 100 µL per tube) for 1 hour at 37°C. Cells were washed with 1 mL of 1X BD Perm/Wash Buffer and stained with BrdU fluorescent anti-BrdU antibody (50 µL, 1:50) for 20 minutes at room temperature. After wash, cells were incubated in 20 µL of the 7-AAD solution and resuspended in 1 mL of staining buffer. The analysis was performed after 1 and 5 days of SF stimulation. Analyses were performed with FACS Aria-III (Becton Dickinson, Franklin Lakes, NJ, USA). Collected data were elaborated by FACS Diva software (Becton Dickinson).

M3) In-Cell Western. Cells were plated in triplicate and stimulated with SF for 24 hours and 5 days. HOXB7 and bFGF presence were assayed by anti-HOXB7 primary antibody (D01P, Abnova) and bFGF (Abcam, ab246354) followed by a secondary antibody conjugated to an infrared dye (IR dye 800 conjugated anti-rabbit IgG; LI-COR Biosciences, Lincoln, NE). Secondary antibody and Cell Tag™ 700 Stain normalizer (LI‐COR Biosciences) were carried out simultaneously. Plates were scanned by Odyssey Infrared Imaging System (LI‐COR Biosciences) in the 800‐nm and 700‐nm channels. Signal was quantified using Odyssey Infrared Imaging System Application Software (LI‐COR Biosciences).
