# Supplementary figures and images for: Microfragmented adipose tissue is associated with improved ex vivo performance linked to HOXB7 and b-FGF expression
Source: Stem Cell Res Ther. 2021 Aug 28;12:481. doi: 10.1186/s13287-021-02540-1 (PMC8399787; doi:10.1186/s13287-021-02540-1)

Fig S1

Lipogems derived MSC

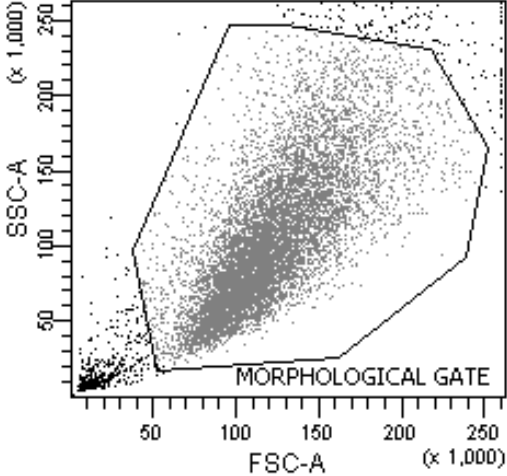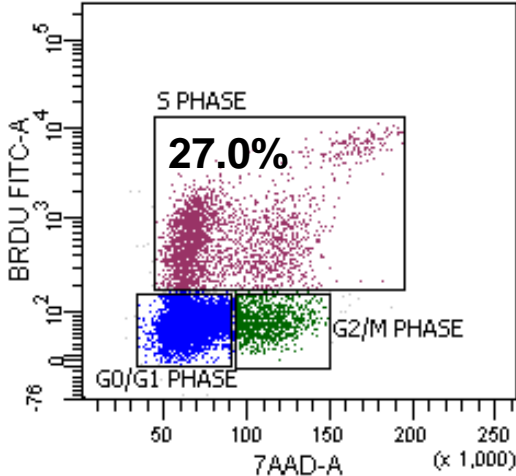

Lipogems derived MSC + SF

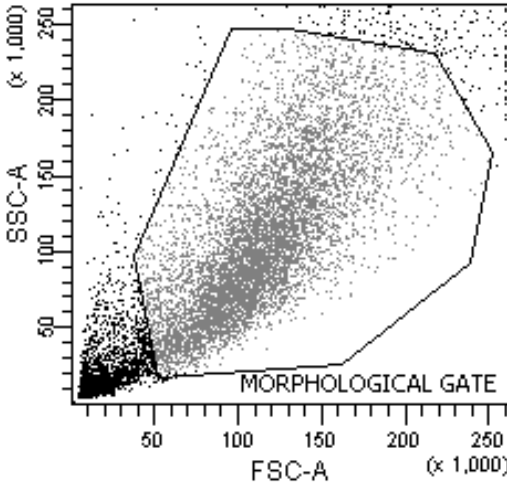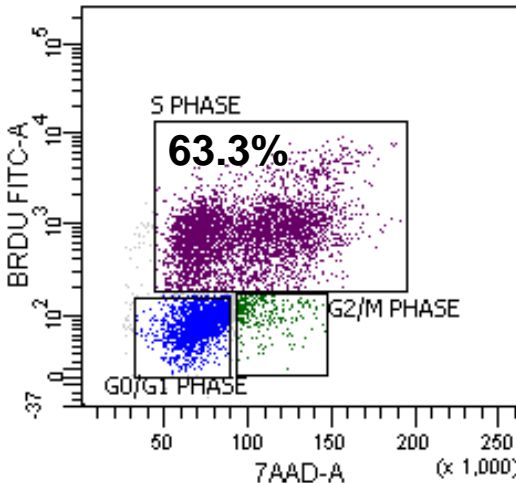

**Fig S2**

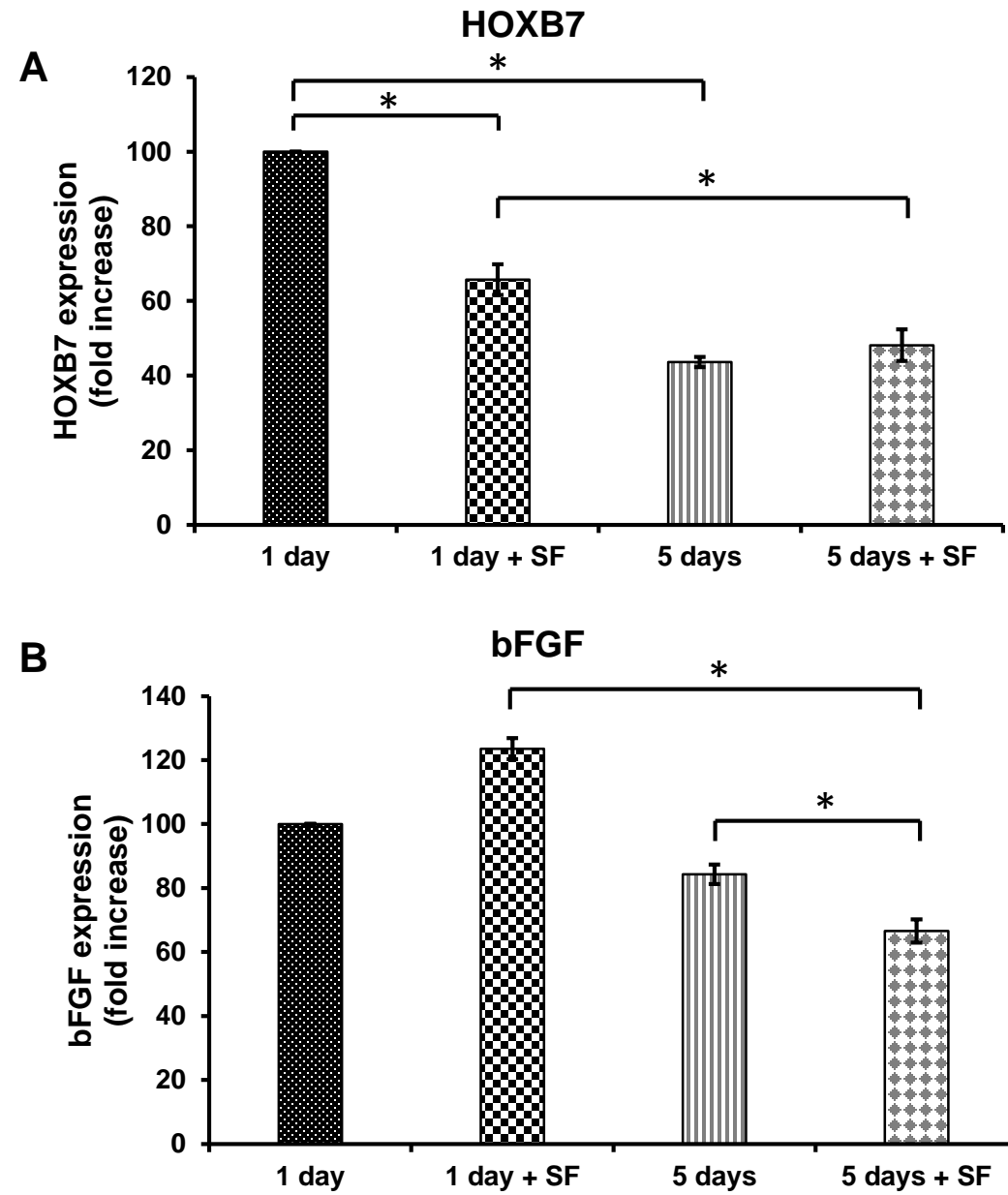

Supplement: Supplementary file 2 — Additional file 2. Fig. S1: BrdU staining of Lipogems derived MSC after SF stimulation for 24 hours. SF stimulation showed approximately twofold increased fluorescence compared to untreated cells, resulting in 63,3% of positive cells when SF is added to cell culture versus 27.0% obtained in untreated cells. Fig. S2: In-Cell Western quantification of the relative intracellular level of HOXB7 (Fig S2A) and bFGF (Fig S2B) protein in Lipogems derived MSC after 1 and 5 days of stimulation with SF. At 1 day of SF stimulation, the level protein of HOXB7 decreases in a statistically significant manner. Indeed, at 5 days no significant effect on HOXB7 protein expression were observed when SF was added. Moreover, untreated cells decreased the levels of HOXB7 at 5 days of culture (p-value<0.05). SF, decreased the level of bFGF after 5 days of culture compared to 1 day of treatment (p-value<0.05). Moreover, bFGF protein decreases when cells are treated with SF for 5 days (p-value<0.05). [file 13287_2021_2540_MOESM2_ESM.pdf]
